# Severe hemorrhage in choriocarcinoma: three scenarios and treatment strategies

**DOI:** 10.1016/j.gore.2025.101930

**Published:** 2025-09-01

**Authors:** J. Altmann, D. Dimitrova, J. Sehouli

**Affiliations:** Department of Gynecology with Center of Oncological Surgery, Charité University Hospital Berlin, Germany

## Abstract

**Introduction:**

Massive hemorrhage originating from the primary tumor site or metastases is a feared complication of choriocarcinoma. We present three cases of severe hemorrhage in patients with gestational choriocarcinoma and their clinical management.

**Case series:**

The first case is a 27-year old woman, who presented with acute hepatic hemorrhage due to metastases of choriocarcinoma 5 months after the birth of her first child. After abdominal packing via laparotomy and transfer of the patient in stable condition to our intensive care unit embolization of several hepatic arteries was performed by interventional angiography. The second case, a 28-year old primipara, presented with hemoperitoneum as a result of uterus perforation by choriocarcinoma. Surgical sutures of the perforated lesion were applied via laparotomy, preserving the uterus. The third case is a 28-year old primigravida with massive vaginal hemorrhage of a choriocarcinoma with pulmonary metastases. Stabilization of the bleeding was achieved by embolization of uterine arteries.

All patients were classified as high-risk (FIGO classification) gestational choriocarcinoma and received EMA/CO chemotherapy until ßHCG reached normal levels as well as up to three additional cycles as consolidation. To date, all patients are in remission.

**Conclusion:**

In case of severe hemorrhage in patients with choriocarcinoma a skilled interdisciplinary team is needed. In case of acute hemorrhage in choriocarcinoma embolization of arteries should be preferred since these are safe and effective measures. In the vast majority of cases fertility-preserving strategies can safely be applied. Hysterectomy is not recommended as first-line treatment.

## Highlights

• In cases of severe hemorrhage associated with choriocarcinoma, management by a skilled interdisciplinary team is essential.
• In the majority of cases, fertility-preserving strategies can be safely implemented.
• Minimally invasive interventions, such as uterine artery embolization, play a critical role in clinical management.

## 1 Introduction

Choriocarcinoma develops in approximately 1 in 50,000 deliveries (Seckl et al., 2010, Seckl et al., 2013). Gestational choriocarcinoma constitutes a distinct entity within the group of gestational trophoblastic neoplasms (GTNs), which also comprises invasive moles, placental site trophoblastic tumor, epithelioid trophoblastic tumor, and mixed trophoblastic tumors (Seckl et al., 2010). These tumors are malignant, ßHCG-producing epithelial neoplasms characterized by central necrosis and a biphasic architecture consisting of cytotrophoblast-like cells and pleomorphic syncytiotrophoblast-like regions (Seckl et al., 2010).

As choriocarcinoma is a highly vascular tumor, massive hemorrhage originating from the primary tumor site or metastases is a serious and potentially life-threatening complication. We present three cases of severe hemorrhage in patients with gestational choriocarcinoma and their clinical management.

## 2 Case series

All three patients provided consent for publication of their cases.

### 2.1 First case

**Fig. 1:**
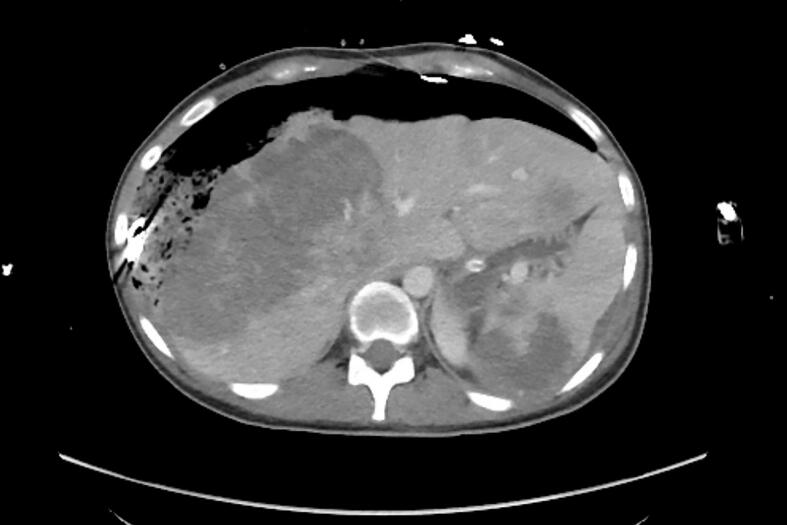
CT scan of the first patient performed after emergency transportation from a different hospital. CT scan demonstrates hepatic metastases and surgical clothes used for abdominal packing to stop hemorrhage from hepatic metastases.

**Fig. 2:**
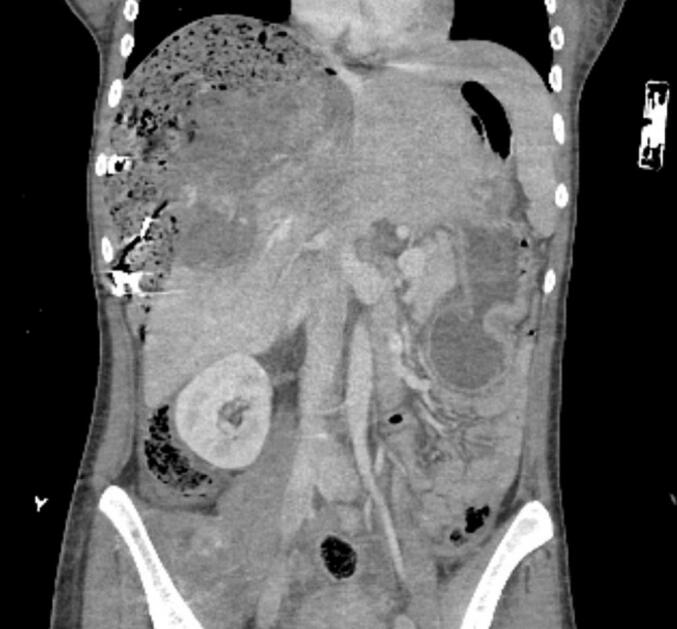
CT scan of the first patient performed after emergency transportation from a different hospital. CT scan demonstrates hepatic metastases and surgical clothes used for abdominal packing to stop hemorrhage from hepatic metastases.

The first case involves a 27-year old woman. In Winter 2022 after being admitted to a regional hospital with upper abdominal pain, she was initially suspected to have acute hepatic hemorrhage due to a hemangioma. She had given birth to her first child 5 months ago. Upon retrospective questioning, she confirmed experiencing metrorrhagia during the postpartum period but was unable to seek medical evaluation due to the time constraints associated with caring for a newborn. After abdominal packing via laparotomy and transfer of the patient in stable condition to our intensive care unit by emergency helicopter (see Fig. 1, Fig. 2).

She was stabilized using adrenaline, fresh frozen plasma and blood units. Immediate angiographic embolization of multiple branches of the middle and right hepatic arteries was performed, followed by re-laparotomy with intrabdominal lavage. On intraoperative inspection, the hepatic lesions appeared highly suspicious for malignancy—either primary or metastatic—and biopsies were obtained to determine the tumor origin. The next day, planned re-laparotomy was performed to remove abdominal packing. Stabilization of hepatic metastases had been achieved successfully.

At the same time, the patient suffered from a thyreotoxic crisis, which was treated with thiamazole, sodium perchlorate, esmolol and hydrocortisone. This was complicated by acute kidney failure and hyperkalemia. Plasmapheresis and hemodialysis were administered. To rule out autoimmune thyroid disorders, autoantibodies including TRAK, TPO, and TG antibodies were tested later and found to be negative. A sonogram revealed normal perfusion and anatomy of the thyroid gland. Given these results, a thyrotoxic crisis due to elevated ßHCG levels was suspected as the underlying cause. Her medication was adjusted to metoprolol, and the hydrocortisone dosage was gradually tapered as her blood pressure and laboratory values normalized.

Biopsy of liver lesions revealed metastatic choriocarcinoma. Human chorionic gonadotropin (HCG) at initial diagnosis was 1 900 000 IU/l. The FIGO score, based on the 2000 FIGO classification system, was 14, indicating a high-risk disease (see Table 1).

**Table 1:** FIGO scores of the three cases.

Prognostic factor	Score											
	0			1			2			4		
Case number	#1	#2	#3	#1	#2	#3	#1	#2	#3	#1	#2	#3
Age (years)	<40			≥40								
	x	x	x									
Antecedent pregnancy (AP) (months)	mole			abortion			term					
							x	x	x			
Interval (end of AP to chemotherapy in months)	<4			4–6			7–12			>12		
		x		x		x						
Pre-treatment hCG (mIU/ml)	<10^3^			>10^3^–10^4^			>10^4^–10^5^			>10^5^		
									x	x	x	
Largest tumor size, including uterus^a^ (cm)	−			3–4			≥5			−		
		x		x		x						
Site of metastases, including uterus	Lung			Spleen, kidney			Gastrointestinal tract			Brain, liver		
		x	x							x		
Number of metastases identified				1–4			5–8					
							x	x	x			
Previous failed chemotherapy	−			−			Single drug			Two or more drugs		
	x	x	x									

The total score for a patient is obtained by adding the individual scores for each prognostic factor. Low risk, 0–6; high risk, ≥7. Stage I, disease confined to the uterus; stage II, disease extending into the pelvis; stage III, disease spread to lungs and/or vagina; stage IV, all other metastatic sites including liver, kidney, spleen and brain.

Adapted from the International Federation of Gynecology and Obstetrics 2025^3^.

FIGO Scores:

Case #1: 14.

Case #2: 8.

Case #3: 8.

a Size of the tumor in the uterus.

Staging imaging via computer tomography (CT) scan revealed metastases of the lungs, liver, spleen, left kidney, the right iliac and obturator muscle.

Given the high-risk classification and the anticipated risk of life-threatening hemorrhage, an multidisciplinary decision was made to initiate induction chemotherapy with cisplatin and etoposide instead of starting standard EMA/CO chemotherapy directly in order to reduce the risk of massive bleeding of hepatic metastases. She received the first cycle of chemotherapy with cisplatin 20 mg/m2 d1-5 q21 and etoposide 100 mg/m2 d1-5 q21dAfterthe patient's general condition has shown marked improvement the chemotherapy regimen was adjusted to EMA/CO, administered every 14 days, with the following doses: etoposide 100 mg/m2 on days 1 and 2, methotrexate 100 mg/m2 intravenous bolus followed by a 12-hour infusion of 200 mg/m2 on day 1, actinomycin D 0.5 mg intravenous on days 1 and 2, calcium folinate 15 mg orally four times on day 2, vincristine 1 mg/m2 intravenous bolus on day 8, and cyclophosphamide 600 mg/m2 intravenous on day 8. Following the 12th cycle of EMA/CO ßHCG values are now within normal ranges. CT scan and MRI 8 months after initial diagnosis show complete remission of liver and spleen metastases, pulmonary metastases are nearly undetectable. Given the extensive distant metastases, the high-risk profile, and the clinical necessity to administer an unusually high number of chemotherapy cycles to achieve normalization of ßHCG levels, the case was thoroughly reviewed with international specialists. A multidisciplinary consensus was reached to initiate off-label therapy with the immune checkpoint inhibitor pembrolizumab. To date, the patient has undergone 24 cycles of 200 mg pembrolizumab administered every three weeks and remains in remission 31 months after the initial diagnosis of metastatic choriocarcinoma.

### 2.2 Second case

The second case is a 28-year old woman. She had given birth to her first healthy child in March.

During the weeks following delivery, abnormal bleeding occurred, leading to uterine curettage being performed at a regional hospital. Surprisingly, histopathology revealed gestational choriocarcinoma rather than the suspected residual placenta. While the histological examination was still in progress, the patient presented with severe abdominal pain and was transferred by emergency helicopter from another regional hospital with a hemoperitoneum.

**Fig. 3:**
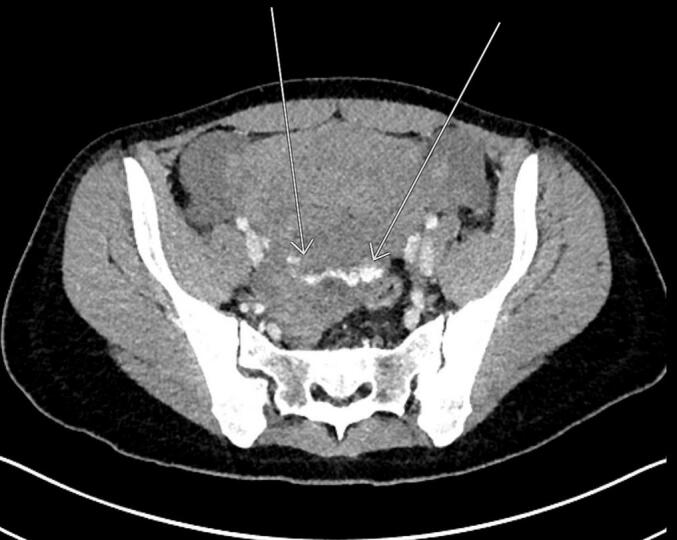
CT scan of the second patient shows diffuse bleeding from the uterus into the abdominal cavity.

**Fig. 4:**
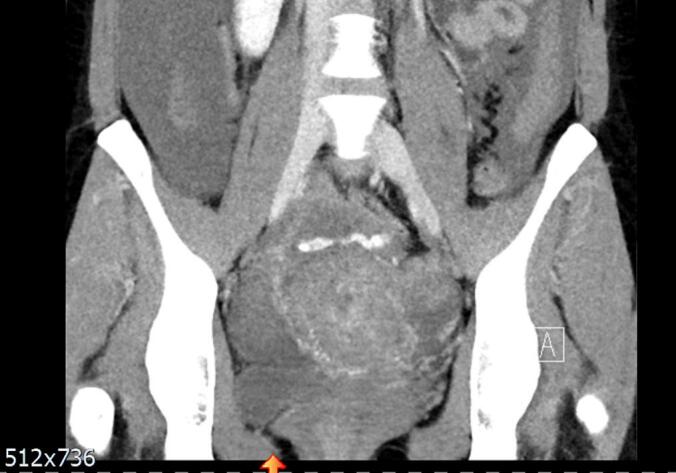
CT scan of the second patient shows diffuse bleeding from the uterus into the abdominal cavity.

She had already received four units of blood before and during transportation. A CT scan was performed at the regional hospital prior to transferring the patient to our facility. Urgent evaluation of the CT scan revealed a tumorous perforation of the uterus, most likely caused by the choriocarcinoma (Fig. 3, Fig. 4). A rapid ultrasound performed in the operating room demonstrated severe hemoperitoneum. The CT scan indicated diffuse bleeding from the uterus into the abdominal cavity. Emergency laparotomy was performed, confirming the diagnosis of uterine perforation due to choriocarcinoma. The lesion was only 1 cm in diameter, but had caused around 4 L of blood loss. We took biopsies were taken from the perforated lesion of the uterine wall as well as via hysteroscopy. The bleeding from the perforated lesion of the uterus was easily stopped by electrocoagulation and suturing, thereby preserving fertility in this patient.

At first presentation to our hospital, ßHCG levels were 170 000 IU/l. Staging via CT scan revealed multiple pulmonary metastases, with the largest measuring 2.1 cm in diameter. Cranial MRI showed no evidence of metastases. Figo score was 8, classifying the patient as high-risk (see Table 1).

Once the patient’s condition had stabilized four days later chemotherapy was initiated with 3 cycles of cisplatin 20 mg/m2 on days 1–5 every 21 days and etoposide 100 mg/m2 on days 1–5 every 21 days. This regimen was followed by 5 cycles of EMA/CO until October. After 3 cycles of EMA/CO ßHCG normalized. Two additional cycles of EMA/CO were administered as consolidation therapy. ßHCG levels were initially monitored weekly, then every 14 days, and are now checked monthly. Regular follow-ups, including gynecological exam and sonography, were performed. At the last follow-up, 28 months after the initial diagnosis, the patient remains recurrence – free.

### 2.3 Third case

The third patient is a 28-year old patient. She gave birth to her third child via cesarean section. Planned cesarean section was performed due to Rhesus incompatibility, abnormal cardiotocography findings and suspicious fetal Doppler sonography results. Following delivery she had experienced abnormal and prolonged bleeding with concomitant anemia. The gynecological exam revealed an increased endometrial thickness, suggestive of placental remnants. After conservative treatment with prostaglandins failed, a curettage was conducted a month after delivery, which histologically confirmed choriocarcinoma. A CT scan identified pulmonary metastases, while cranial MRI was negative for metastases or other abnormalities. The patient’s FIGO score was 8, placing her in the high-risk category (see Table 1).

**Fig. 5:**
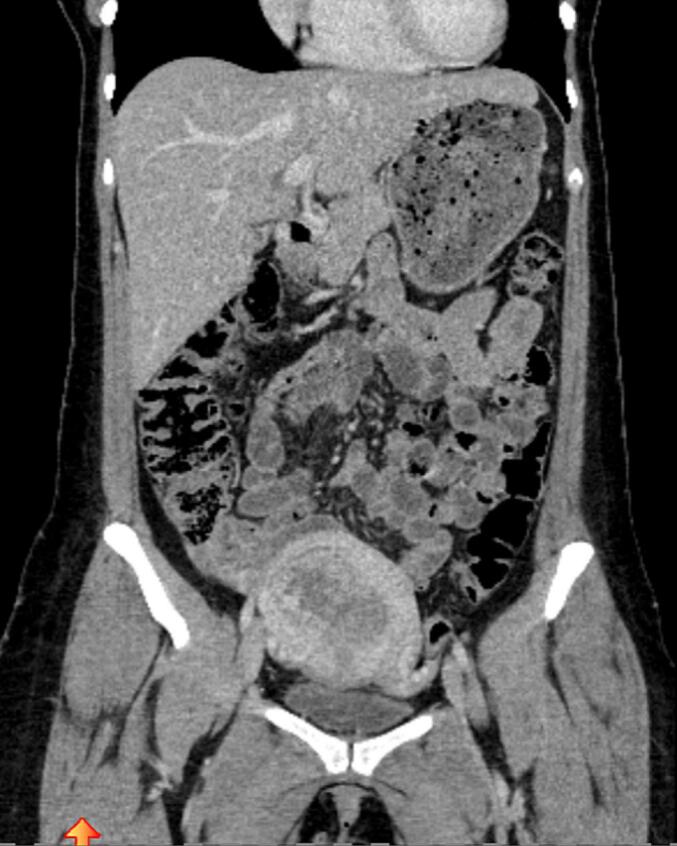
CT scan of the third patient right after severe vaginal bleeding started on the second day of EMA/CO chemotherapy.

**Fig. 6:**
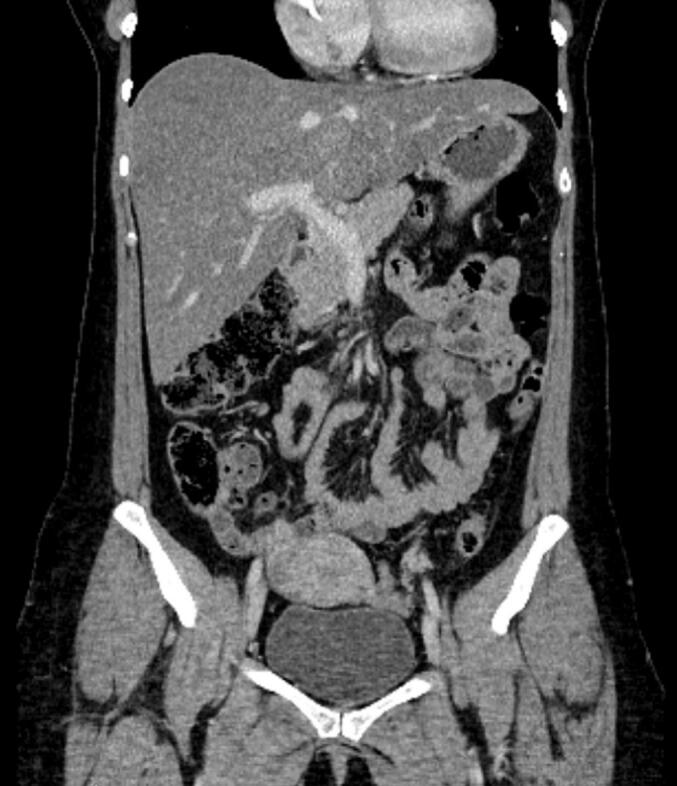
CT scan of the third patient 4 months after embolization of several branches of both uterine arteries.

She was admitted to our ward after histological diagnosis was confirmed with vaginal bleeding and received a port system first. EMA/CO chemotherapy was started. However, on the second day of chemotherapy, she began to experience uncontrolled vaginal bleeding (Fig. 5). Cardiovascular stabilization was achieved by administration of electrolytes, blood and plasma transfusions, and tranexamic acid under intensive care unit (ICU) supervision. Blood transfusion was complicated by the patient́s rhesus negative blood type with irregular heat antibodies, so optimal blood bags were requested from other hospitals in the region and transported to our clinic specifically for this patient. This was followed by emergency particle and gel embolization of several side branches of both uterine arteries. After embolization she was observed at the ICU for three days in stable condition (Fig. 6). Testing for several coagulation disorders revealed no abnormalities. Chemotherapy with EMA/CO was continued at regular intervals of 14 days. Additionally, the patient presented with pain in her arm and a fresh thrombosis of the port system of the right vena subclavian was diagnosed. Therapeutic anticoagulation was administered.

In total, she received 7 cycles of EMA/CO, three of which were administered as consolidation therapy after normalization of ßHCG levels. No grade 3 or 4 complications were observed. During the last follow-up 19 months after initial diagnosis, the patient is still in remission and regular follow-ups including gynecological sonography and HCG values are performed. A regular 28 day cycle was reported by the patient.

## 3 Discussion

The cases presented above highlight the complexities involved in managing very high-risk gestational choriocarcinoma. Establishing a diagnosis of gestational choriocarcinoma can be difficult, as the disease may manifest months or even years after a prior pregnancy or abortion. Initial staging imaging should include body CT, brain MRI, pelvic MRI, and Doppler ultrasonography (Ngan et al., 2003). Staging in our patients demonstrated hepatic metastases in case 1 and pulmonary metastases in cases 2 and 3.

Measurement of β-hCG is crucial, as the diagnosis of choriocarcinoma is typically associated with abnormally high β-hCG levels inconsistent with normal pregnancy. In addition, β-hCG serves as a valuable marker for treatment monitoring, enabling early detection of drug-resistance or recurrence (Seckl et al., 2010, Seckl et al., 2013). As reported above, all cases recorded extremely high β-hCG levels, with values exceeding 10^4^ in case 3 and surpassing 10^5^ in cases 1 and 2. Treatment response was evaluated through both β-hCG measurements and radiological imaging in our cases.

The FIGO 2000 scoring system stratifies gestational trophoblastic disease into low- and high-risk categories (Bolze et al., 2015) (see FIGO scores of the presented cases in Table 1). While treatment with methotrexate or actinomycin D is effective in low-risk GDT, polychemotherapy with EMA/CO (Ngan et al., 2003, Bogani et al., 2023) consisting of etoposide, methotrexate, and actinomycin D (EMA), alternating weekly with cyclophosphamide and vincristine (CO), is recommended for high-risk disease (Seckl et al., 2010, Seckl et al., 2013). Five-year overall survival following EMA/CO chemotherapy is reported to be approximately 86 % (Bogani et al., 2023). Adverse prognostic factors include the presence of liver or brain metastases, a long interval since the antecedent pregnancy, and term delivery of the antecedent pregnancy (Bogani et al., 2023).

Notably, the first case presented with life-threatening hemorrhage from liver metastases of gestational choriocarcinoma, and a FIGO very high-risk classification, making it by far the most complex case to manage.

After normalization of ß-hCG levels chemotherapy should be continued for six weeks, or for eight weeks if poor prognostic features such as liver or brain metastases are present (Seckl et al., 2010, Seckl et al., 2013).

The risk of disease progression during or after primary chemotherapy is around 20 % in high-risk gestational trophoblastic neoplasia (GTN) patients; however, approximately 75 %–80 % of these patients can still be successfully salvaged (Bower et al., 1997).

As the cases described above were classified as high-risk, multi-agent chemotherapy with EMA/CO was administered successfully resulting in complete remission in all patients.

### 3.1 Management in case of severe hemorrhage

Choriocarcinoma (CC) is known to be a highly invasive, vascular and metastatic tumor (Seckl et al., 2010, Seckl et al., 2013). Due to its extensive vascularization, there is a significant risk of life-threatening hemorrhage.

Although chemotherapy has replaced surgery as the treatment of choice for gestational trophoblastic tumors, nearly 50 % of patients with high-risk GTN (FIGO stages II–IV, score > 7) require surgical intervention during the course of therapy to remove disease or manage complications. Surgical procedures may be performed to: (1) excise resistant or persistent lesions in the uterus or metastases, (2) reduce tumor burden in the uterus, (3) control hemorrhage, (4) relieve bowel or urinary obstruction, or (5) treat infections (Alifrangis et al., 2013, Lurain et al., 2006, Lurain, 2010). Management of severe hemorrhage in choriocarcinoma requires a skilled multidisciplinary team of gynecologists, surgeons, anesthesiologists and (interventional) radiologists. First, stabilization of the patient’s vital functions must be ensured through intensive care measures, blood and plasma transfusions, monitoring of coagulation, accurate measurement of blood loss, and preparation of subsequent steps to control the bleeding (Alifrangis et al., 2013). Second, a comprehensive therapeutic approach, considering all available medical and surgical options, should be discussed.

Historically, hysterectomy was frequently performed in cases of severe bleeding. However, contemporary management prioritizes an individualized – if possible fertility sparing – approach. Today, minimal-invasive techniques such as angiographic arterial embolization often provide an alternative. Embolization of tumor-feeding vessels have proven to be both safe and effective, reducing the need for radical surgery, whether applied as a standalone procedure or prior to surgical intervention (Lurain, 2010).

Still, laparotomy may be needed to stop bleeding in organs such as the liver, gastrointestinal tract, kidneys, and spleen (Alifrangis et al., 2013, Lurain et al., 2006, Lurain, 2010, Ngu and Ngan, 2021). Lurain et al also described cases of suturing of the liver or uterus for bleeding within their cohort of patients with high-risk gestational trophoblastic neoplasia (Alifrangis et al., 2013).

In two of the above described cases particle embolization was used, of hepatic arteries to arrest bleeding from hepatic metastases of choriocarcinoma in case one and of uterine vessels to stop bleeding from intrauterine choriocarcinoma in case three. In a case series by Lim et al hemorrhage was controlled in the majority of GTD patients with bleeding of residual uterine vascular malformations via embolization with a common femoral artery approach (Lim et al., 2002). After angiographic embolization five pregnancies leading to three full-term deliveries were achieved in this study population of 11 women (Lim et al., 2002).

In settings where minimally invasive options are unavailable, urgent patient transfer to a specialized center should be considered, provided that adequate stabilization can be achieved. As illustrated by the presented cases, emergency laparotomy with abdominal packing may also serve as a lifesaving interim measure.

In the above mentioned third patient, severe hemorrhage developed during the first cycle of EMA/CO chemotherapy. In this case, given the absence of bleeding and the patient’s good general health, EMA/CO therapy was started immediately after histological confirmation. However, during the course of treatment, she experienced sudden, heavy vaginal bleeding. This highlights the significant risk of hemorrhage during chemotherapy from rapid tumor lysis and shrinkage, exposure and then rupture of neoplastic vessels under cytotoxic therapy. According to the ESMO guidelines, to reduce early deaths in patients with very advanced disease due to severe bleeding, it is recommended to commence chemotherapy gently with low-dose etoposide 100 mg/m^2^ and cisplatin 20 mg/m^2^ on days 1 and 2 repeated weekly for 1–3 weeks (Seckl et al., 2010, Seckl et al., 2013). This therapeutic regimen can be combined with dexamethasone 24 mg in 24 h to reduce tumor oedema. Low-dose induction etoposide and cisplatin has helped to improve long-term overall survival data to over 94 % in high-risk patients (Ngan et al., 2003). We followed this recommendation in the above mentioned first two cases as severe hemorrhage had already occurred. In both cases, chemotherapeutic regimen was adjusted to EMA/CO after 3 cycles. In the third case EMA/CO was started immediately but it likely provoked severe hemorrhage. Retrospectively, considering our clinical experience now, induction chemotherapy would rather be the preferred approach for cases similar to the third patient.

### 3.2 Fertility-conserving

Fertility-conserving is possible in the vast majority of cases. Hysterectomy as primary treatment strategy is not recommended. Also, removal of residual masses is unnecessary since it does not reduce risk of recurrence, which is less than about 3 %^1^. Fortunately, fertility is not otherwise affected with 83 % of women becoming pregnant after either MTX/FA or EMA/CO chemotherapy. However, EMA/CO brings forward the menopause date by 3 years (Seckl et al., 2010). Moreover, there is no obvious increase in the incidence of congenital malformations (Seckl et al., 2010).

Since the risk of relapse after chemotherapy is around 3 % and most occur within the first year of follow-up, pregnancy should ideally be delayed until beyond this period. Frequent monitoring of hCG for at least 12 months with reliable contraception is recommended (Seckl et al., 2010, Seckl et al., 2013, Ngan et al., 2025). During hCG follow-up, patients are encouraged to use reliable contraception, any method of contraception can be used, although practitioners usually refrain from using oral contraceptives until hCG levels are within normal ranges.

If a patient becomes pregnant, in-depth sonographic evaluation of the fetus, placenta and uterus is recommended. After delivery return of ßHCG levels to normal should be ensured.

### 3.3 Thyreotoxicity

In the first case, a rare complication of choriocarcinoma in the form of a thyrotoxic crisis occurred. Thyrotoxic crisis is an extremely rare condition (Saleem et al., 2021, Kofinas et al., 2015), with neurological manifestations including altered mental status, agitation, psychosis, seizures, and coma. Gastrointestinal manifestations may include jaundice, nausea, vomiting, and abdominal pain (Saleem et al., 2021). In the context of choriocarcinoma, this condition may be related to elevated levels of β-hCG (Saleem et al., 2021, Kofinas et al., 2015, Kato et al., 2004), which show enhanced thyrotropic activity and structural similarity to TSH, leading to thyroxine release from the thyroid gland (Saleem et al., 2021, Kofinas et al., 2015, Kato et al., 2004). Studies suggest that higher β-hCG levels are associated with an increased risk of thyrotoxicosis (Saleem et al., 2021). Notably, the first patient exhibited extremely high β-hCG levels of 1,900,000 IU/L. Treatment of thyrotoxic crisis includes thiamazole or carbimazole, β-blockers, and corticosteroids, while carefully monitoring neurological status and blood pressure.

### 3.4 Risk of drug-resistance or recurrence

According to the literature, approximately 20–35 % of high-risk patients will fail first-line therapy or relapse from remission. Most of these patients will have a clinicopathologic diagnosis of choriocarcinoma, a large tumor burden reflected by a high hCG level and multiple metastases to sites other than the lung and pelvis, resulting in very high FIGO scores (Alifrangis et al., 2013). Hysterectomy might be appropriate in carefully selected cases where the disease is drug-resistant and confined to the uterus. In two studies, 70–86 % of patients who had hysterectomy for resistant choriocarcinoma survived (Alifrangis et al., 2013, Pisal et al., 2002).

Fortunately, to date, all three patients remain tumor-free and are under close follow-up monitoring.

## 4 Conclusion

In case of severe hemorrhage in patients with choriocarcinoma a skilled interdisciplinary team gynecologists, anesthesiologists and (interventional) radiologists is needed. An individualized approach incorporating minimally invasive procedures, including arterial embolization of tumor-feeding uterine or metastatic vessels, may be employed either as a standalone measure or before surgery, should be considered. In the vast majority of cases fertility-preserving strategies can safely be applied. Hysterectomy is not recommended as first-line treatment.

## Declaration of competing interest

The authors declare that they have no known competing financial interests or personal relationships that could have appeared to influence the work reported in this paper.
